# Suicide after Leaving the UK Armed Forces —A Cohort Study

**DOI:** 10.1371/journal.pmed.1000026

**Published:** 2009-03-03

**Authors:** Navneet Kapur, David While, Nick Blatchley, Isabelle Bray, Kate Harrison

**Affiliations:** 1 Centre for Suicide Prevention, University of Manchester, Manchester, United Kingdom; 2 Defence Analytical Services and Advice (Ministry of Defence), Bath, United Kingdom; King's College London, United Kingdom

## Abstract

**Background:**

Few studies have examined suicide risk in individuals once they have left the military. We aimed to investigate the rate, timing, and risk factors for suicide in all those who had left the UK Armed Forces (1996–2005).

**Methods and Findings:**

We carried out a cohort study of ex-Armed Forces personnel by linking national databases of discharged personnel and suicide deaths (which included deaths receiving either a suicide or undetermined verdict). Comparisons were made with both general and serving populations. During the study period 233,803 individuals left the Armed Forces and 224 died by suicide. Although the overall rate of suicide was not greater than that in the general population, the risk of suicide in men aged 24 y and younger who had left the Armed Forces was approximately two to three times higher than the risk for the same age groups in the general and serving populations (age-specific rate ratios ranging from 170 to 290). The risk of suicide for men aged 30–49 y was lower than that in the general population. The risk was persistent but may have been at its highest in the first 2 y following discharge. The risk of suicide was greatest in males, those who had served in the Army, those with a short length of service, and those of lower rank. The rate of contact with specialist mental health was lowest in the age groups at greatest risk of suicide (14% for those aged under 20 y, 20% for those aged 20–24 y).

**Conclusions:**

Young men who leave the UK Armed Forces were at increased risk of suicide. This may reflect preservice vulnerabilities rather than factors related to service experiences or discharge. Preventive strategies might include practical and psychological preparation for discharge and encouraging appropriate help-seeking behaviour once individuals have left the services.

## Abstract 2

Navneet Kapur and colleagues find that young men who leave the United Kingdom Armed Forces are at increased risk of suicide.

## Editors' Summary

**Background.:**

Leaving any job can be hard but for people leaving the armed forces the adjustment to their new circumstances can sometimes be particularly difficult. For example, ex-military personnel may face obstacles to getting a new job, particularly if they were injured in action. Some become homeless. Others turn to alcohol or drugs or suffer mental illnesses such as depression. These things probably aren't common but those who leave the armed forces might also be at higher risk of suicide than the general population.

**Why Was This Study Done?:**

Serving members of the UK Armed Forces (the British Army, the Naval Service, and the Royal Air Force) have a lower rate of suicide than the general UK population. The lower rate is probably due to “the healthy worker effect” (i.e., workers tend to be healthier than the general population, since the latter includes people unable to work due to illness or disability). However, there are anecdotal reports that ex-military personnel are more likely to die by suicide than are members of the general population. If these reports are correct, then measures should be put into place to prepare people for leaving the Armed Forces and to provide more support for them once they have left the military. The authors of this new study say that no previous studies had systematically examined suicide risk in individuals leaving the Armed Forces. In this new study, therefore, the researchers examine the suicide rate, timing, and risk factors for suicide in a large group (cohort) of former members of the UK Armed Forces.

**What Did the Researchers Do and Find?:**

The researchers linked data on everyone who left the UK Armed Forces between 1996 and 2005 with information on suicides collected by the National Confidential Inquiry into Suicide and Homicide. Since 1996, the Inquiry has been collecting information about all suicides (defined as cases where the coroner has given a verdict of suicide or of “undetermined death”) in the UK, including information about whether the deceased used mental health services in the year before they died. The aim of the Inquiry is to reduce the risk of suicides (and homicides) in the UK by improving the country's mental health services. Between 1996 and 2005, 233,803 people left the Armed Forces and 224 (nearly all men) died by suicide. The researchers' statistical analysis of these data indicates that the overall suicide rate in the ex-military personnel was similar to that in the general population. However, the risk of suicide in men aged 24 y or younger who had left the military was 2–3 times greater than that in the same age group in both the general male population and in men serving in the Armed Forces. The risk of dying by suicide was highest in the first 2 y after leaving the military but remained raised for several years. Risk factors for suicide among ex-military personnel included being male, serving in the Army, having a short length of service, and being of lower rank. Only a fifth of the ex-military personnel who committed suicide had been in contact with mental health services in the year before they died, and the rate of contact with these services was lowest among individuals in the age groups at the highest risk of suicide.

**What Do These Findings Mean?:**

These findings indicate that young men leaving the UK Armed Forces are at increased risk of suicide, particularly shortly after leaving. The study was not able to prove the reason for this increased risk, but the authors suggest three main possibilities: (1) the stress of transitioning to civilian life, (2) exposure to adverse experiences while in the military, or (3) a vulnerability to suicide before entering the military. The study provides some evidence to support the third hypothesis—untrained personnel with short lengths of service have a particularly high risk of dying by suicide after leaving the military, suggesting that the increased suicide risk may reflect a pre-military vulnerability. The researchers suggest that practical and psychological preparation might be helpful for people leaving the Armed Forces and that appropriate help-seeking behavior could be encouraged in these individuals. In the UK, the National Health Service is currently piloting a community-based mental health service for military veterans, characterized by regional clinical networks involving partnerships of relevant experts.

**Additional Information.:**

Please access these Web sites via the online version of this summary at http://dx.doi.org/10.1371/journal.pmed.1000026.

- This study is further discussed in a *PLoS Medicine* Perspective by Jitender Sareen and Shay-Lee Belik
- The Manchester University Centre for Suicide Prevention provides information about the National Confidential Inquiry into Suicide and Homicide and about other research into suicide, and a list of useful Web sites and help lines for people going through crises
- A recent article in the Observer newspaper by Mark Townsend discusses the problems facing UK military personnel when they leave the Armed Forces
- Information about suicides among serving members of the UK Armed Forces is published by the Defence Analytical Services Agency
- The UK National Health Service provides information about suicide, including statistics about suicide in the UK and links to other resources
- MedlinePlus also provides links to further information and advice about suicide
- The World Health Organization provides information on the global burden of suicide

## Introduction

The health of ex-service personnel continues to attract significant public interest [1]. For a minority the transition to civilian life is difficult [2]. It may result in social exclusion, homelessness, alcohol misuse, unemployment, and poor mental health [3,4]. Suicide is an important cause of premature mortality in the general population [5]. Although there have been anecdotal reports of increased suicide risk in specific groups of veterans [6], no studies to our knowledge have systematically examined suicide risk in individuals once they leave the military.

The rate of suicide among those serving in the UK Armed Forces has been reported to be lower than that in the general population and this is likely to reflect a “healthy worker effect” (the phenomenon of lower morbidity or mortality in certain occupational groups compared to the general population because those with severe illness or disability are less likely to be employed in those occupations) [7]. A number of studies have examined suicide outcomes in relation to recent conflicts. For example, in the UK, researchers found no differences in suicide mortality between those deployed to the 1990–1991 Gulf War and a matched cohort of individuals who were serving in the Armed Forces but were not deployed [8,9]. These findings are largely consistent with US studies of individuals who served in the same conflict [10,11]. A further group of studies from Scandinavia have examined suicide in those who have served as peacekeepers. These individuals may not be typical of full-time regular personnel and findings have been inconsistent [12,13]. One US study examined outcomes over 12 y in a cohort of individuals who indicated on a population survey that they had served in the military at any time [14]. The study reported that those who had served in the Armed Forces were twice as likely to die by suicide as those who had not served. However military service was indicated by self-report, the study sample was heterogeneous (ranging from those who had served in World War I to those who had served in the post-Vietnam era), and the general population comparison group was much younger than the ex-service cohort.

In general, there has been very little research internationally investigating suicide in those who have left the Armed Forces and studies have tended to focus on those who have served in specific conflicts (a proportion of whom remain in service). Because suicide is a comparatively rare outcome, most studies have included few such deaths and have not examined suicide risk in relation to age. Equally, there has been no exploration of the time elapsed since discharge and little consideration of the risk factors for suicide in relation to leaving the military. Although previous work has suggested that some ex-service personal may be reluctant to seek help for mental health problems [15], no studies to our knowledge have examined rates of contact with mental health services prior to suicide. We sought to overcome these difficulties by linking discharge data with national suicide data in order to carry out a study of suicide risk in all individuals who left the UK Armed Forces over a 10-y period. We had four specific objectives: to investigate the rate of suicide in those who had left the UK Armed Forces; to investigate the timing of suicide in this group in relation to the time elapsed since discharge; to identify potential risk factors for suicide; to explore the rates of contact with mental health services prior to suicide and describe the characteristics of this service-contact group.

In addition, we wished to compare the rate of suicide and rates of contact with mental health services prior to suicide in those who had left the Armed Forces with those serving in the Armed Forces.

## Method

### Design

We carried out a retrospective cohort study of ex-Armed Forces personnel and our main outcome was death by suicide after leaving the services. Comparisons were made with both general and serving populations. A case-control analysis was carried out on the subset of this cohort in contact with mental health services in the 12 mo prior to death.

### Setting and Individuals

This national study covered the whole of the UK. We included individuals who had left any of the three branches of the Armed Forces (British Army, Naval Service [including Royal Navy and Royal Marines], Royal Air Force [RAF]) between 1st April 1996 and 31st December 2005 with no restriction on length of military service (that is, we included anyone who left after their first day of basic training). Reservists who are deployed may have worse mental health outcomes postdeployment than regulars [16], but we excluded reservists from this study because of the lack of consistently available discharge data, comparatively small numbers of expected suicide deaths, and the fact that reservists are a heterogeneous group. We also excluded personnel who served in the Gurkha Regiment who were easily identifiable from the databases and would in general return to Nepal after discharge. Their deaths would be unlikely to be recorded on UK national databases.

### Database of Those Who Had Left the Armed Forces

This database was compiled by the Defence Analytical Services Agency (DASA) primarily from data held by the Manpower Branches of each of the three services. These data were checked for consistency against historical information stored by DASA and overall numbers were compared with those reported in UK Defence Statistics. The resulting database represented the best available data on those discharged from the UK Armed Forces. We included a limited number of core variables and data were over 90% complete for these.

### Suicide Databases

We used the databases held by the National Confidential Inquiry into Suicide and Homicide by People with Mental Illness to identify deaths by suicide during the study period (April 1996–December 2005). This study period was chosen because the Confidential Inquiry commenced its data collection in April 1996, and 2005 was the last year for which complete data were available. The Inquiry holds both a general population suicide database (which includes all suicide deaths in the UK) and a more detailed clinical database of those who have been in contact with specialist mental health services in the 12 mo prior to death.

The Inquiry's general population database receives data from the Office for National Statistics (ONS) for England and Wales, and the General Register Offices (GRO) for Northern Ireland and Scotland. These are the definitive sources for general population suicide data in the respective countries. Individuals who receive a verdict of suicide (ICD 10 Codes X60-X84 and Y87.0) or undetermined death (“open verdict”) (ICD 10 Codes Y10-Y34 [excluding Y33.9 verdict pending and Y87.2]) at inquest were included in the sample and are referred to as cases of suicide in the remainder of the paper. Excluding open verdicts may result in an almost 50% underestimate in the number of suicide deaths [17]. Official suicide statistics in the UK are also based on this wider definition of suicide (that is, suicide and open verdicts), as are in-service suicide statistics [7]. The general population database includes details of age, gender, and method of suicide. The Inquiry's patient database includes detailed demographic and clinical information on all those who have had contact with specialist National Health Service (NHS) mental health services in the 12 mo prior to suicide. The Inquiry methods have been described in detail elsewhere [18,19].

### Database Linkage

We linked the database of those who had left the Armed Forces with the suicide databases using last name, first name (where available), date of birth, and gender. Using the approximately 70% of individuals for whom we had full first names we were able to determine the optimal matching strategy for the remaining 30% of individuals for whom we had just initials (see Text S1). We checked the robustness of our linking procedures by investigating how many of the UK sample of in-service suicide deaths identified by DASA during the study period were also present on the suicide databases used in this study. We were able to successfully match 163/177 (92%) of UK suicide deaths.

### In-service Suicide Data

Data on suicide deaths (including both suicide and undetermined deaths) among all serving personnel during the study period were provided by DASA.

### Ethical and Other Approvals

The protocol for this study was scrutinized by the North West Multi-Centre Research Ethics Committee (MREC) and was judged not to require additional health ethics approval. The study was also approved by the Ministry of Defence Ethics Committee (MOD(N)PREC).

### Analysis

In order to compare rates to the UK general population, we calculated age-specific mortality ratios using 5-y age bands and an overall standardised mortality ratio (SMR). Simple descriptive statistics were used to characterise the sample and we calculated crude suicide rates by time since leaving the Armed Forces. We investigated which factors measured at discharge were associated with subsequent suicide by using survival analysis (generating hazard ratios [HRs] using Cox's proportional hazards models). We modelled the effects of age and length of service as both categorical and continuous variables. The Inquiry patient database was used to determine the rate of contact with mental health services in the 12 mo prior to death. In order to compare the characteristics of those who had died within 12 mo of contact in the leavers' sample with those who had died in an appropriate general population comparison group we selected up to five controls per case matched on age (within 1 y), gender, and year of death from the Inquiry patient database and carried out a conditional logistic regression analysis. Five controls per case gave us 80% power to examine relative risks of 2.5 and above (assuming a prevalence of a risk factor of 30% in the civilian comparison group and α = 0.05). We used denominators supplied by DASA to determine the crude rate of suicide in serving personnel per 100,000 strength. Finally in order to compare rate of suicide in the leavers' sample with the rate in serving personnel, we calculated age-specific mortality ratios and SMRs (standardised in this instance, to the serving rather than the general population).

## Results

### Characteristics of the Discharged Cohort and Those Who Died by Suicide

In total we obtained data on 233,803 individuals who had left the UK Armed Forces between 1st April 1996 and 31st December 2005 (representing over 98% of all those who left during this period). These individuals accumulated a total of 1,159,194 person years at risk during the study period with a mean follow up period of 5 y. The median age (interquartile range [IQR]) of the cohort at discharge was 25 y (20–34 y) and 210,175 (90%) were male. Overall 137,021 (59%) had served in the Army, 50,818 (22%) in the Naval Service, and 45,964 (20%) in the RAF. Approximately 7% of the sample (*n* = 15,329) were listed as being discharged because of medical reasons, but it should be noted that the protocols for medical discharge vary by service.

Overall, 224 individuals were found to have died by suicide after leaving the Armed Forces, on the basis of linkage between the discharge and Inquiry databases. Their median age (IQR) was 22 y (19–29 y) and they were predominantly male (215 [96%]). Overall 163 (73%) had served in the Army, 34 (15%) in the Naval Service, and 27 (12%) in the RAF. Hanging or strangulation (99 cases [44%]) and self-poisoning (47 cases [21%]) were the most common methods of suicide. Deaths involving firearms occurred in only five cases (2%). Methods of suicide were generally similar to those in the general population during this time period, although hanging deaths were slightly more common in the discharged cohort compared to the general population (44% versus 37%) and self-poisoning was slightly less common in the discharged cohort (21% versus 27%). These differences may be a reflection of the different age and gender composition of the discharged cohort compared with the general population. The median time to death (IQR) after leaving the services was 31 mo (16–57 mo).

### Rate of Suicide

Table 1 shows the crude rate of suicide, age-specific mortality ratios, and the SMR for males who left the Armed Forces. Although overall the rate of suicide was not greater than that in the general population (SMR [95% confidence interval (CI)] 97 [84–110]), the risk of suicide in the two youngest age groups was approximately two to three times higher than in same age groups in the general population (age-specific mortality ratios of 170 [134–213] in those aged 20–24 y and 293 [185–441] in those aged under 20 y). For males aged 30–49 y the age-specific mortality ratios suggested that the risk of suicide was lower than for the same age groups in the general population, although the upper limit of the 95% CIs were close to 100 in some cases.

**Table 1:**
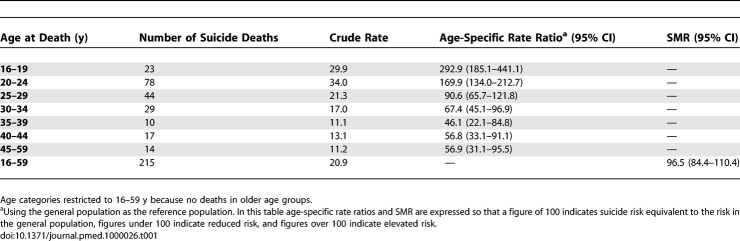
Numbers, Crude Rates per 100,000 Person Years, and Age-Specific Rate Ratios for Suicide in Males Who Left the UK Armed Forces 1996–2005

An analysis of female deaths was limited by very small numbers but showed an overall SMR that was not significantly elevated (SMR [95% CI] 133 [69–255]). However there was an increased risk of suicide in the youngest female age group, although the CIs were wide because of small numbers (age-specific mortality ratio [95% CI] for those aged under 20 y: 860 [177–2,529]).

The pattern of suicide mortality was similar across services with the highest risks generally in the youngest age groups. Comparatively small numbers of suicide deaths in the Naval Service and RAF samples meant that the elevated age-specific mortality ratios for males observed in the youngest age groups were not always statistically significant (age-specific mortality ratios [95% CI] for males aged <20 y: Army 279.5 [162.4–449.0]; Naval Service 361.6 [117.2–845.7]; RAF 258.3 [6.5–1,441.4]). Age-specific mortality ratios (95% CI) for males aged 20–24 y: Army 191.5 [148.1–243.8]; Naval Service 88.8 [35.7–183.1]; RAF 132.3 [36.0–339.0]). The overall SMR was significantly elevated for the Army only (SMR [95% CI] 127 [108–148]).

With respect to period effects, splitting the data by years (1996–2000 versus 2001–2005) and adjusting the male SMR for time period made little difference to the overall findings (adjusted SMR [95% CI] 100 [87–114]).

### Timing of Suicide

**Figure 1:**
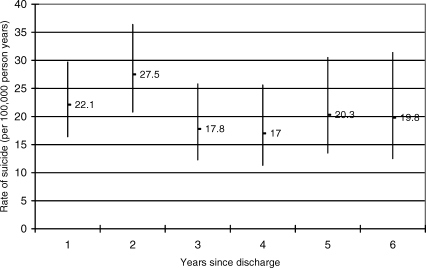
Rate of Suicide (and 95% CI) in Those Who Left the UK Armed Forces by Time Elapsed Since Discharge

Figure 1 shows the rate of suicide by time elapsed since discharge. Due to small numbers these data are presented for males only. Risk appeared to be greatest in the first 2 y following discharge—rate of suicide per 100,000 person years (95% CI): 24.7 (20.1–30.2) in the first 2 y after discharge versus 18.7 (15.7–22.4) for follow up periods of greater than 2 y. Although the risk of suicide was at its highest in the early post discharge period, it was to some extent persistent—rate of suicide per 100,000 person years in the sixth year after discharge (95% CI): 19.8 (12.5–31.4).

### Risk Factors for Suicide

Suicide risk was associated with younger age at discharge, male gender, Army Service, lower rank, untrained status, not being married, and length of service of 4 y or less (Table 2). After adjustment for service branch (Army, Naval Service, RAF) all these associations remained statistically significant with the exception of training status. Further adjustment for age made little difference to the risk factors.

**Table 2:**
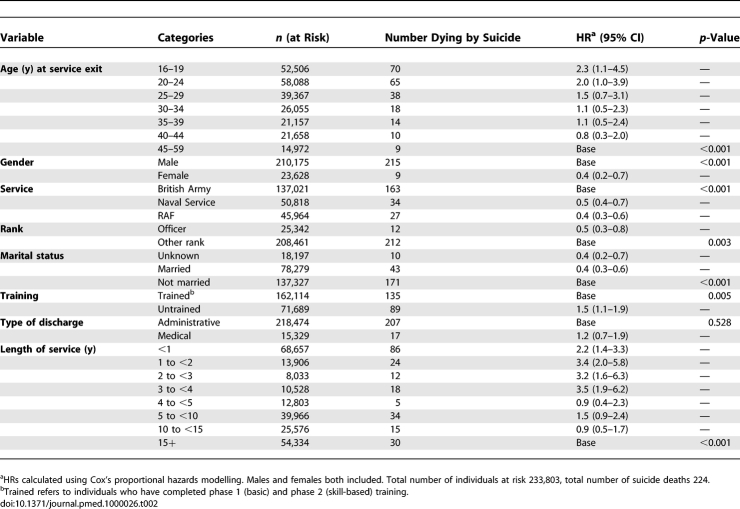
Risk Factors for Suicide

Two additional models that utilised the properties of age as a continuous predictor were also fitted. First, the effect of age on suicide risk was modelled using one linear term. This produced a HR (95% CI) of 0.96 (0.95–0.98), i.e., a drop in risk of 4%, year on year from age 16 y. Second, age was modelled using two linear terms. From age 16 to 45 y the HR (95% CI) was 0.95 (0.93–0.97) and from age 46 y upwards the HR (95% CI) was 1.01 (0.99–1.02), i.e., a drop in risk of 5% year on year from age 16 to 45 y and then a nonsignificant increase in risk of 1% year on year from age 46 y. The cut off of 45 y was used because a preliminary examination of the data suggested the risk might change at this point. With respect to length of service in relation to suicide risk, a single linear term produced a HR of 0.96 (0.94–0.97) i.e., a drop in risk of approximately 4% for each additional year of service.

Two variables in particular were likely to be strongly related to age at discharge—length of service and training status. Length of service of less than 4 y (compared to length of service of 4 y or longer) was significantly associated with risk of suicide after adjustment for age at discharge (HR [95% CI] 2.1 [1.4–3.1]). Being untrained (compared to being trained) was no longer significantly associated with suicide (HR [95% CI] 0.9 [0.6–1.3]).

### Rates and Characteristics of Those in Mental Health Service Contact Prior to Suicide

Of the 224 individuals who died by suicide after leaving the Armed Forces, 47 (21%, 95% CI 16%–27%) had been in contact with mental health services in the year before death, a slightly lower proportion than had been in contact with mental health services in the year before suicide in the general population (28%, 95% CI 27.5%–28.2%). Although numbers were small, the proportion of those who had left the Armed Forces and had contact with mental health services prior to suicide was lowest in the youngest age groups (14% in those aged under 20 y, 20% in those aged 20–24 y). Table 3 compares the characteristics of those in contact with mental health services prior to death in the discharged cohort with individuals matched for age, gender, and year of death who were also in contact with mental health services prior to death but had not served in the Armed Forces. Those who had left the forces were more likely to have a history of alcohol misuse. They were rated as at lower long-term risk of suicide by clinicians and were less likely to have had contact with mental health services in the week prior to death.

**Table 3:**
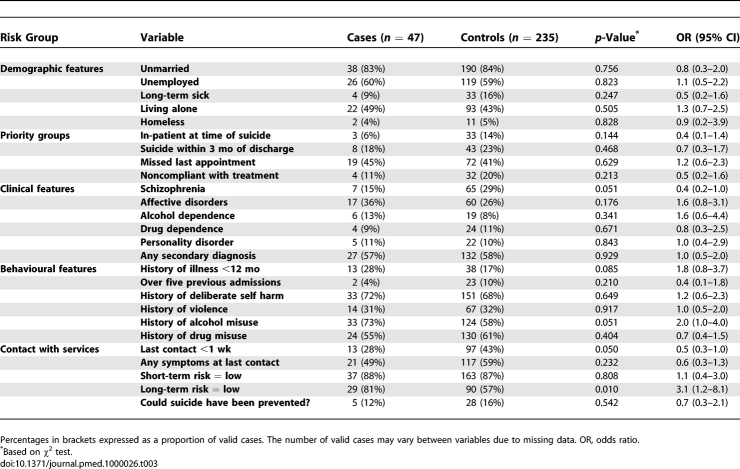
Characteristics of Individuals Who Left the Armed Forces and Who Had Contact with Mental Health Services in the 12 Mo Prior to Suicide and Matched Controls Who Had Not Served in the Armed Forces

### Comparison with Inservice Cohort

There were 222 suicide deaths in the serving sample during the study period. Table 4 presents crude rates of suicide for males who left the Armed Forces and crude rates of suicide for males serving in the Armed Forces during the study period. In this table, the age-specific mortality ratios and SMR compare the observed rate of suicide in those who left the Forces to the expected rate (if those who left had the same suicide rate as serving personnel). The data suggest that the overall risk of suicide is twice as high following discharge than in-service, with elevated risks in all age groups under 30 y. The overall SMR for females who left the Armed Forces was also somewhat elevated (age-specific rate ratios not shown) but because of comparatively small numbers the CIs were wide and the overall SMR nonsignificant (SMR [95% CI] 183 [64–524]).

**Table 4:**
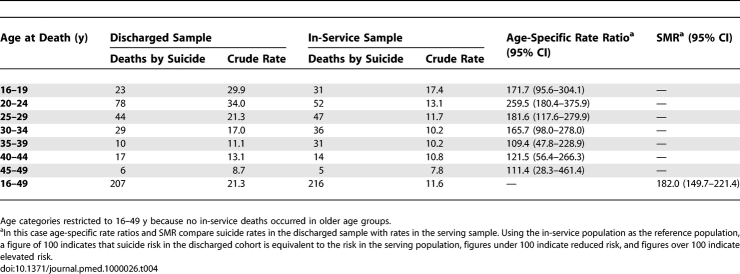
Numbers and Crude Rates per 100,000 for Suicide in Males Who Left the UK Armed Forces and Males Serving in the Armed Forces 1996–2005

## Discussion

### Main Findings

We found that the risk of suicide in men aged 24 y and younger who had left the Armed Forces was approximately two to three times higher than the risk for the same age groups in both the serving and general populations. The risk was persistent but may have been at its highest in the first 2 y following discharge. The risk of suicide was greatest in males, those who had served in the Army, and those with a short length of service. Lack of training and lower rank were also associated with suicide risk, although associations with training status became insignificant after adjustment for service or age. The rate of contact with specialist mental health services in the 12 mo prior to death was comparatively low at 21% (compared to 28% for the general population) and lowest in the age groups at greatest risk of suicide (14% for those aged under 20 y, 20% for those aged 20–24 y).

### Methodological Issues

This study was the first to our knowledge to systematically investigate suicide risk in an unselected sample of individuals once they had left the Armed Forces. It was a comparatively large cohort that enabled examination of suicide rates in different age groups, the timing of deaths, and risk factors for suicide. By linking to definitive sources of national data we can be confident that we had reasonably complete ascertainment of outcomes.

However, our findings need to be interpreted in the context of a number of methodological shortcomings. Our matching procedures were robust, but for 30% of the discharged sample we had initials as opposed to first names. We were able to devise an optimal matching strategy. Had we accepted a weaker matching strategy (matches on surname, date of birth, gender, and any initial), this would have resulted in a greater number of possible false positive matches, and the number of suicide deaths in those who had left the Armed Forces would have increased to 291. The resulting overall SMR for males (standardised to the general population) would indicate an elevated risk of suicide that was now significant (SMR [95% CI] 126 [112–142]). The overall SMR for females would remain elevated but nonsignificant (SMR [95% CI] 131.5 [70.7–244.3]).

We used UK data sources and we may have missed some cases of suicide among discharged personnel that occurred overseas. However, this may not have been a major problem. A previous study of over 100,000 service personnel suggested less than 1% left the UK over an 8-y follow up period [8].

We used the general population as our main comparison group. It would not have been feasible to select a reference population matched for pre-enlistment socioeconomic factors. Instead we compared the leavers' cohort to the age-matched general population but also the age-matched serving population.

We used administrative databases to obtain information on those who had left the Armed Forces and we were not able to explore the role of some potentially important variables in later suicide risk including deployment history, physical and psychological injuries, and mental health status prior to entry to the military.

With respect to deployment, large cohort studies of UK and US veterans of the 1991 Gulf War have not found an effect of deployment or experiences during deployment on subsequent mortality [8–11]. A report on psychiatric morbidity in the UK Armed Forces (based on attendance to military community mental health departments) found no difference in overall rates of mental disorder between those who had been deployed and those who had not been deployed [20]. A recent study of US Armed Forces personnel returning from Iraq and Afghanistan reported no overall increase in suicide mortality for the cohort as a whole compared to the general population, but the authors did find a slightly higher than expected rate of suicide in those who saw active service [21].

Physical injury during service may also affect the risk of suicide. In a US study of over 30,000 individuals who had served in Vietnam, those individuals who were wounded on more than one occasion and admitted to hospital were nearly twice as likely to die by suicide as the general population [22]. Other studies have suggested that the psychological sequelae of combat (for example, post-traumatic stress disorder [PTSD]), although uncommon, may increase the risk of suicide relative to the risk in those without any psychiatric diagnosis [23].

No studies have specifically examined post-service suicide risk in relation to mental health status prior to entry to the military. Studies have suggested that prerecruitment factors such as poor family relationships, problematic behaviours, or negative life events may influence the risk of suicidal behaviour [24,25].

Potentially important antecedents such as relationship breakdown or legal or financial problems could not be examined in this study. These factors might best be investigated in future studies using a “psychological autopsy” design where detailed information is collected through interviews with informants (for example close relatives, or professionals involved in care) [25].

The use of administrative databases meant that for some variables (for example, rank, type of discharge) we were only able to use a relatively crude dichotomous categorisation. We were unable to look at the reasons for discharge in more detail or look separately at individuals who left before their terms of enlistment were completed. When we excluded those who left within the first year of service, the overall SMR for males was reduced slightly (81.4 [95% CI 68.7–96.5]) but the age-specific rate ratios for males remained elevated in the two youngest age groups (<20 y: 455.3 (95% CI 123.9–1168.2). 20–24 y 234.6 (95% CI 166.6–321.4).

### Interpretation of Findings

How might we interpret the high rate of suicide in younger individuals after they have left the Armed Forces? There are three main possibilities. The first is that leaving the Armed Forces and the transition to civilian life may be extremely difficult for some individuals. The second explanation relates to in-service exposure—those with the highest risk of suicide after discharge may have had the most adverse experiences while they were in the military. The third possibility is that high suicide risk is a consequence of premilitary vulnerability. The current study is unable to conclusively distinguish between these hypotheses, but does provide some potentially useful supporting evidence.

Some authors have highlighted the potential problems in going from what might be perceived as a highly ordered institutional environment to a relatively unstructured civilian life [26]. Had this been the sole explanation for the increased suicide risk we might have expected to see a more marked gradient in risk over time. We might also have expected to see the greatest risk associated with the longest length of service but this was not the case. We did not aim to examine the role of adverse in-service exposures in determining postdischarge suicide risk in this study and so are unable to draw any firm conclusions about the importance of these factors. There is some evidence that pre-service vulnerability is associated with negative health outcomes [24,25]. Consistent with the vulnerability hypothesis are our findings that young, untrained individuals with short lengths of service were at greatest risk of suicide after discharge.

What is clear is that younger age groups were at risk of suicide but rates of contact with mental health services were low. Those who had left the Armed Forces had higher rates of alcohol misuse but clinicians rated them as at lower risk and they were less likely to have been seen by mental health services in the week before death. These findings could reflect difficulties in the accessibility or acceptability of NHS mental health services to those who have left the Armed Forces or the difficulties NHS services may have in assessing the treatment needs of veterans [15].

One possibility we were unable to explore in detail in this study was the protective effect of serving in the Armed Forces. It should be borne in mind that military service might prevent suicide in some individuals. We found that the age-specific rates of suicide in discharged personnel aged 30–49 y were lower than the rates in the same age groups in the general population. There is evidence to suggest that serving may have a positive effect on a variety of outcomes such as resilience, employment, and socioeconomic attainment [2,27].

### Implications

Young people who left the UK Armed Forces were at high risk of suicide. These findings are likely to have relevance to other Western countries with professional Armed Forces but further studies in different settings are needed to confirm this finding. Whatever the explanation for our findings, these individuals may benefit from some form of intervention.

Initial prerecruitment interview, medical examination, high induction standards, and training are obviously important in ensuring a healthy military [28], but it should be recognised that those who are selected out of service at any of these stages may be at potentially high risk of adverse outcomes including suicide.

What form might interventions take? The current study was observational in nature and was not designed to provide evidence for the efficacy of interventions. The main strategies used in the UK to date have been practical and psychological preparation for discharge [26,29] and encouraging appropriate help-seeking behaviour once individuals have left the Armed Forces [4]. All those who receive medical discharges are currently entitled to a full resettlement package and there are also initiatives designed to help early service leavers access appropriate services after they have left [30]. Some authors have pointed out that other countries (for example the USA and Australia) have dedicated health care systems for veterans [4]. However in the UK, the centrally funded health system, the focus on social inclusion, and the comparatively small numbers of veterans, have meant that the emphasis is on providing treatment within the NHS. Recently the UK Department of Health wrote to all Primary Care, Acute, and Mental Health Trusts emphasising that veterans should receive priority access to secondary care for any conditions that were likely to be service-related [31]. In addition a community-based mental health service for veterans led by the NHS and characterised by regional clinical networks involving partnerships of relevant experts is currently being piloted [32]. Voluntary sector organizations have also played a role in both raising awareness of possible poor outcomes and providing services for those who have left the Military [33].

## Supporting Information

Text S1 Database Linkage and Matching Strategy (25 KB DOC)

## Abbreviations

CI: confidence interval
DASA: Defence Analytical Services Agency
HR: hazard ratio
IQR: interquartile range
NHS: National Health Service
RAF: Royal Air Force
SMR: standardised mortality ratio
