# Supplementary material for: Suicide after Leaving the UK Armed Forces —A Cohort Study
Source: PLoS Med. 2009 Mar 3;6(3):e1000026. doi: 10.1371/journal.pmed.1000026 (PMC2650723; doi:10.1371/journal.pmed.1000026)
Supplement: Supplementary file 1 [file pmed.1000026.sd001.doc]

The databases were formatted to ensure consistency. We linked the database of those who had left the Armed Forces with the suicide databases using last name, first name (where available), date of birth, and gender. Using the approximately 70% of individuals for whom we had full first names we were able to determine the optimal matching strategy for the remaining 30% of individuals for whom we had just initials. Cases were initially matched on last name, date of birth and gender. Accepting matches on all 3 initials, all 2 initials, 2 out of 3 initials, or one initial (where only one initial was listed on the database) but rejecting matches on 1 of 2 or 1of 3 initials identified a very high proportion of first name matches (over 90%) with very few false matches (less than 5%). This was therefore the strategy we used to match individuals where initials (rather than full first names) were available. In some cases individuals were known by more than one name (for example, if names had been changed following marriage and for other reasons). We checked all available names and also checked alternative spellings and name endings (for example Mc, Mac, and Mack; Smith, Smyth and Smythe). All potential matches were verified by manual examination by one of the research team (DW), and any uncertain matches referred to a second rater (NK). We checked the robustness of our linking procedures by investigating how many of the UK sample of in-Service suicide deaths identified by DASA during the study period were also present on the suicide databases used in this study. We were able to successfully match 163/177 (92%) of UK suicide deaths. Once data linkage was complete, names and Service numbers were stripped from the database.
